# Bridging gaps and cultivating care: a call for culturally competent dermatological education for ethnic hair

**DOI:** 10.1097/JW9.0000000000000192

**Published:** 2025-01-16

**Authors:** Jane Onyemachi, Elise Weisert, Lindy Ross

**Affiliations:** a John Sealy School of Medicine, The University of Texas Medical Branch, Galveston, Texas; b Department of Dermatology, The University of Texas Medical Branch, Galveston, Texas

**Keywords:** cultural competency, dermatology education, ethnic hair, hair camouflage techniques, hair disorders

**What is known about this subject in regard to women and their families?**

- There is a recognized gap in dermatological education concerning the unique hair care needs of African American women. Studies have highlighted the lack of training dermatologists receive in managing hair loss and conditions prevalent in this population.
- Additionally, certain hair care methods, such as braids, weaves, and wigs, come with potential complications like traction alopecia and allergic reactions. This underscores the necessity for dermatologists to be well-versed in these methods to provide safe and effective care.

**What is new from this article as messages for women and their families?**

- This letter proposes the development of formalized continuing medical education programs for dermatologists. This program aims to bridge the educational gap by familiarizing practitioners with hair camouflage techniques and their implications for hair health. Such an initiative could significantly enhance patient satisfaction and improve health outcomes by ensuring that dermatologists provide care that is culturally informed and respectful.
- The call for integrating cultural competence into dermatological practice is a key message for women and their families. It highlights the need for dermatologists to understand and respect cultural hair care practices, which can lead to better health outcomes and more personalized care.
- This letter emphasizes that improving dermatological education for African American hair care is not just beneficial for individual patient encounters but also contributes to broader systemic improvements in healthcare equity. By advocating for culturally competent care, the article aligns with the ongoing efforts to address healthcare disparities and promote inclusive medical practices.

## Dear Editors,

The recent article by Taye et al.^1^ initiates an essential dialogue on the intersection of dermatological practices and cultural hair styling within the African American community. This publication is not only timely but also crucial, as it addresses a longstanding educational deficiency in dermatology that lags behind the demographic realities of our patient populations. Recent studies have highlighted the urgent need for culturally attuned dermatological care, especially regarding hair loss treatments in populations with unique hair styling practices.^2,3^

The comprehensive discussion in the article about hair camouflage techniques, from wigs and extensions to innovative approaches like micropigmentation, illuminates not only the diversity of solutions but also the necessity for dermatologists to master these methods comprehensively. The potential for these techniques to exacerbate conditions such as traction alopecia or cause allergic reactions underscores the need for dermatologists to have a thorough understanding of their application and implications.

In response, we propose the development of a formalized continuing medical education (CME) program specifically for dermatologists and dermatologists in training. This program would not only familiarize practitioners with a spectrum of hair camouflage techniques but also keep them abreast of evolving methodologies and their implications for hair health. Implementing such a program could significantly enhance patient satisfaction and improve health outcomes by ensuring that dermatologists can provide care that respects and understands cultural practices associated with African American or ethnic hair.^4,5^

To address the educational gap in dermatological training on ethnic hair, we propose the development of an online learning module in collaboration with organizations such as the Skin of Color Society (SOCS) and the American Academy of Dermatology (AAD). Ideally, this module would be delivered online and qualify for CME credit. Alternatively, it could be presented at academic conferences within individual institutions, as well as at local and national dermatology conferences.

This program would be developed in collaboration with dermatologists specializing in hair disorders, as well as cultural competence experts and stylists experienced in ethnic hair care. SOCS, with its focus on the education and care of patients with skin of color, and AAD, with its curriculum dedicated to skin of color, would be ideal partners in increasing awareness about hair camouflage techniques and their implications for hair health. By working with these organizations, the module would ensure accuracy, relevance, and cultural sensitivity, reaching not only dermatologists but also dermatology residents, physicians, and nonphysician providers who oversee the care of patients with alopecia or other hair loss disorders.

Accrediting this program for CME credit is critical to making it accessible and attractive to practicing dermatologists. The online module could be hosted on platforms such as the AAD’s Learning Center^6^ or SOCS’s Dermatology E-Learning + Equity Platform.^7^ This would allow easy access for dermatologists, dermatology residents, and other healthcare providers seeking to enhance their understanding of hair disorders in patients with ethnic hair. Offering CME credits would incentivize participation and help ensure dermatologists remain up-to-date on these critical issues.

The proposed module would consist of several components:

(1) Introduction to common hair disorders:
This section will provide a brief overview of conditions disproportionately affecting African American patients, such as traction alopecia, central centrifugal cicatricial alopecia, and folliculitis decalvans. The inclusion of dermoscopic findings within the curriculum will facilitate a more comprehensive understanding of these conditions.
(2) Culturally sensitive hair care practices:
Training on the cultural significance of hair in the African American community, with a focus on common styling practices (eg, braids, weaves, wigs) and the potential dermatological consequences of some of these practices.
(3) Overview of hair camouflage techniques:
This section will focus on the various hair camouflage methods commonly employed, such as wigs, weaves, braids, micropigmentation, and the use of products like hair fibers and topical concealers to camouflage thinning hair, and the role of dermatologists in advising patients on their safe use.
(4) Management and prevention strategies:
Evidence-based approaches for addressing alopecia and preventing further hair loss in patients with ethnic hair, with a focus on early intervention and patient education.
(5) Interactive case-based learning:
Scenarios that challenge participants to identify various hair camouflage methods and engage patients in culturally competent, sensitive discussions about alopecia. The goal of these case-based learning scenarios is to allow participants to apply their knowledge in a practical, low-risk setting, addressing not only the physical aspects of hair loss but also the emotional and cultural dimensions that are critical to providing comprehensive patient care.
(6) Assessing and addressing practice gaps:
A key component of the proposed online learning modules involves identifying and addressing current practice gaps in providing care for African American patients with alopecia and other hair disorders. These gaps often arise due to limited familiarity with the unique presentation of hair disorders in patients with ethnic hair and insufficient understanding of culturally specific hair practices, which may contribute to hair loss or serve to camouflage it.

One of these gaps includes the lack of knowledge surrounding appropriate treatment options that account for the specific needs of ethnic hair. Choosing suitable treatments and medications is essential for effective management, particularly when considering the diverse textures and conditions that affect patients with ethnic hair. For instance, traditional treatments for conditions like seborrheic dermatitis in Caucasian patients, such as ketoconazole shampoo, may be too drying for individuals with ethnic hair. Providers should be advised that it is important to either apply such treatments directly to the scalp or avoid them altogether when moisture retention is a priority.^8^ Additionally, consideration of formulation is crucial, as patients with ethnic hair may prefer ointment or oil-based formulations that help retain moisture, whereas Caucasian patients may prefer gel or foam formulations.^8^

These subtle adjustments can enhance patient compliance and treatment success, while also strengthening the patient–physician relationship by aligning care with the specific needs of ethnic hair care.

By offering this module as an online CME course or as a presentation at academic conferences, we aim to establish a sustainable and scalable approach to educating dermatologists on ethnic hair care. This platform would provide dermatologists with deeper insights into the significant emotional, cultural, and social impacts of alopecia within the African American community. With a heightened awareness and sensitivity, dermatologists may build stronger, more trusting relationships with their patients, leading to improved treatment adherence and ultimately better health outcomes.

As the field of dermatology continues to evolve, it is imperative that it adapts to meet the needs of a diverse patient base, ensuring that all patients receive care that is respectful, informed, and effective. The development of such specialized online learning modules is crucial, with benefits extending beyond individual patient encounters to broader systemic improvements in healthcare equity.

## Conflicts of interest

None.

## Funding

None.

## Study approval

N/A

## Author contributions

JO contributed to the conception and drafting of the letter. EW and LR provided critical revisions. All authors approved the final version.

## Acknowledgments

The authors would like to acknowledge Bianca Uzoma, MD for her mentorship with this manuscript.
